# Observation of intermediate states of the human prion protein by high pressure NMR spectroscopy

**DOI:** 10.1186/1472-6807-6-16

**Published:** 2006-07-17

**Authors:** Norman Kachel, Werner Kremer, Ralph Zahn, Hans Robert Kalbitzer

**Affiliations:** 1 Institut für Biophysik und Physikalische Biochemie, Universität Regensburg, Universitätsstr. 31, 93053 Regensburg, Germany; 2 Institute of Molecular Biology and Biophysics, ETH Hönggerberg, CH-8093 Zürich, Switzerland; 3 Alicon AG, Wagistrasse 23, 8952 Zürich-Schlieren, Switzerland

## Abstract

**Background:**

Prions as causative agents of transmissible spongiform encephalopathies (TSEs) in humans and animals are composed of the infectious isomer, PrP^Sc^, of the cellular prion protein, PrP^C^. The conversion and thus the propensity of PrP^C ^to adopt alternative folds leads to the species-specific propagation of the disease. High pressure is a powerful tool to study the physico-chemical properties of proteins as well as the dynamics and structure of folding intermediates.

**Results:**

Conformational intermediates of the human prion protein *hu*PrP^C ^were characterized by a combination of hydrostatic pressure (up to 200 MPa) with two-dimensional NMR spectroscopy. All pressure effects showed to be reversible and there is virtually no difference in the overall pressure response between the folded core of the N-terminal truncated *hu*PrP^C^(121–230) and the full-length *hu*PrP^C^(23–230). The only significant differences in the pressure response of full-length and truncated PrP suggest that E168, H187, T192, E207, E211 and Y226 are involved in a transient interaction with the unfolded N-terminus. High-pressure NMR spectroscopy indicates that the folded core of the human prion protein occurs in two structural states N_1_and N_2 _in solution associated with rather small differences in free enthalpies (3.0 kJ/mol). At atmospheric pressure approximately 29% of the protein are already in the pressure favored conformation N_2_. There is a second process representing two possible folding intermediates I_1 _and I_2 _with corresponding average free enthalpies of 10.8 and 18.6 kJ/mol. They could represent preaggregation states of the protein that coexist at ambient pressure with a very small population of approximately 1.2% and less than 0.1%. Further the pressure response of the N-terminus indicates that four different regions are in a fast equilibrium with non-random structural states whose populations are shifted by pressure.

**Conclusion:**

We identified pressure stabilized folding intermediates of the human prion protein. The regions reflecting most strongly the transition to the intermediate states are the β1/α1-loop and the solvent exposed side of α3. The most pressure-sensitive region (representing mainly intermediate I_1_) is the loop between β-strand 1 and α-helix 1 (residue 139–141), indicating that this region might be the first entry point for the infectious conformer to convert the cellular protein.

## Background

Transmissible spongiform encephalopathies (TSEs), or prion diseases, are infectious fatal disorders of the central nervous system (CNS) which include Creutzfeldt-Jakob disease, Gerstmann-Sträussler-Scheincker syndrome, fatal familial insomnia, and kuru in humans, bovine spongiform encephalopathy in cattle, scrapie in sheep, and chronic wasting disease in deer and elk [1]. They are associated with the accumulation of an oligomeric conformational scrapie isomer, PrP^Sc^, of the host-encoded monomeric prion protein PrP^C ^[2]. According to the "protein only" hypothesis, PrP^Sc ^is the sole component of transmissible prions [3]. One of the main supports for this hypothesis is the finding that PrP knockout (*PRNP*^0/0^) mice are completely protected against scrapie disease and fail to propagate prions [4,5] and that introduction of murine *PRNP *transgenes into these mice restores the susceptibility to prions [6]. The formation of PrP^Sc ^involves a conformational switch by which PrP^C ^is transformed into the PrP^Sc ^conformation with PrP^Sc ^as template [7]. Recently Legname *et al*. succeeded in their attempt to form infectious PrP^Sc ^from recombinant PrP^C ^without the use of infectious material, which strongly supports the "protein only" hypothesis [8]. Puzzling to this hypothesis is the existence of a wide variety of distinct so-called prion strains with differing infectivity and related to them the mechanism of the species barrier, i.e., the degree of the interspecies infectivity of a prion strain [9]. Primarily, the differences in PrP^Sc ^amino acid sequence related to heterology of the PrP genotype may account for the formation of distinct prion strains and for the species barrier [10]. For this reason hamster prions are usually not infectious to wild-type mice and there is a moderate species barrier for transmission of mouse prions to hamster. However, formation of distinct prion strains is even known, when the strains have identical primary structure and the hosts are genetically identical [11]. In this sense a recent article of the Prusiner group [12] provides evidence that PrP^Sc ^conformation and species barrier may be independent of the infectious isomer and the degree of homology between the PrP of the donor and that of the host. The species barrier should better be defined as the inability of the host to replicate the conformation of the infectious isomer and is broken only by altering the original and generating a conformational distinct isoform [12]. The fact that prion strains are encoded in the conformation of the protein has recently been proven for yeast prions [13,14]. Several lines of evidence suggest that this is also true for mammalian PrP, as reviewed by Chien *et al*. [15]. More recently Vanik *et al*. [16] showed that sequence-based barriers that prevent cross-seeding between prion proteins from different species can be bypassed *in vitro *by a template-induced adaptation process leading to the emergence of new strains of prion fibrils. Structural work should elucidate and identify the properties that determine the species barrier and the reproducibility or lack of reproducibility of a prion strain in a given host.

Structures of the cellular prion proteins from different species are known, among these hamster [17,18], human [19-21] and bovine [22]. Acidic pH can induce a scrapie-like unfolding intermediate of the prion protein [23] and was shown to convert the human prion protein reversibly between native monomeric and fibrilogenic conformations [24]. The application of hydrostatic pressure is known to stabilize folding intermediates and was applied to the study of aggregates and amyloids [25-28]. Recently a number of groups performed high pressure experiments on mammalian prion protein [29-33]. The combination of high hydrostatic pressure with high resolution NMR spectroscopy allows the observation of pressure induced structural changes and shifts of conformational equilibria as well as local instabilities of the protein at atomic resolution. Kuwata *et al*. [34] used high pressure NMR spectroscopy to stabilize a locally disordered conformer of the hamster prion protein.

Here we report results from high pressure NMR spectroscopy on two isoforms of the human prion protein, *hu*PrP(121–231) and *hu*PrP(23–231), at pH 4.8 and 293 K in the pressure range from 0.1 to 200 MPa. The application of pressure potentially allows detecting structural intermediates of PrP which may be important for the transformation process between cellular and infectious scrapie-type protein.

## Results

### General pressure effects on full length and truncated human prion protein

1D ^1^H- and 2D ^1^H-^15^N-TROSY NMR spectra of two different constructs of the ^15^N enriched human prion protein, *hu*PrP(23–230) and *hu*PrP(121–230), were recorded at pH 4.8 (acetate buffer) at various pressures. We used solution conditions identical to those used for the structure determination [19]. Particularly for *hu*PrP(23–230) slightly acidic pH is necessary because the protein aggregates at neutral pH [35]. The shorter construct *hu*PrP(121–230) contains essentially the C-terminal folded part of the protein, the longer construct *hu*PrP(23–230) in addition the unfolded N-terminus of the prion protein. Under theses conditions they showed nearly identical and completely reversible pressure responses in the range from 0.1 to 200 MPa. At 293 K we applied hydrostatic pressures in steps of 0.1, 50, 100, 125, 150, 175 and 200 MPa to both, *hu*PrP(23–230) and *hu*PrP(121–230). Increasing pressure results in chemical shift changes of all resonances. In addition, in the TROSY-HSQC spectra the increased pressure leads to a decrease of the signal volumes of a number of resonances. Some of the signals disappear completely from the spectra while others show no significant volume change as described below (see section "pressure response of cross peak volumes").

### Pressure dependence of the chemical shifts in the truncated and full length prion protein

In general, the pressure response of proteins is anisotropic and the chemical shift changes induced by the local and global conformational changes are a non-linear function of the pressure. Traditionally, the pressure dependence is described by the zero-order coefficient δ_0_(*p*_0_, *T*_0_), the first order coefficient B_1 _and the second order coefficient B_2 _of a Taylor expansion around the pressure *p*_0 _and the temperature *T*_0 _(see eq. 1). When the pressure effects are assumed to be composed of two contributions, an unspecific effect as observed in random-coil peptides and a specific, structure dependent effect, then the influence of these effects on the Taylor coefficients can be assumed as additive as long as the two events are not or only weekly coupled (see Methods). In this case, the corrected, conformation dependent pressure (Taylor) coefficients B_1_* and B_2_* can be obtained by subtracting the corresponding pressure coefficients from random-coil peptides. Random-coil pressure coefficients are available for the ^1^H-shifts from a study of the model peptides Gly-Gly-X-Ala peptides [36] but not for the ^15^N-shifts.

**Figure 1:**
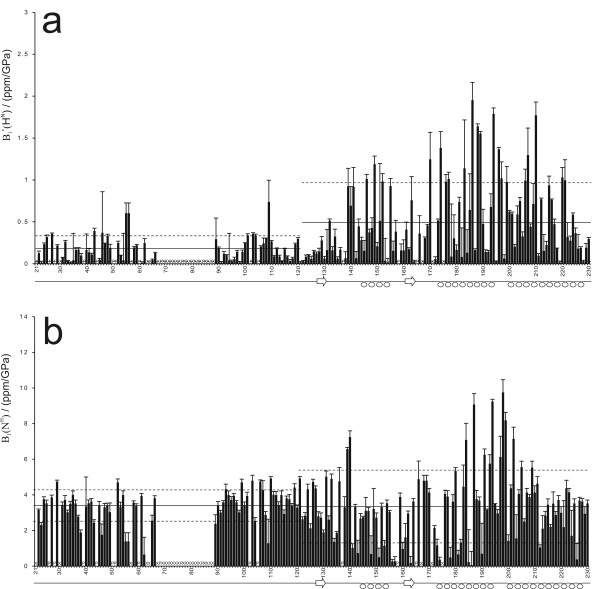
First order pressure coefficients of the ^1^H^N ^and of the ^15^N^H ^chemical shifts taken from the TROSY-HSQC spectra of *hu*PrP(23–230). Spectra were measured at 293 K at 600 MHz proton frequency. The absolute values of the conformation dependent pressure coefficients |B_1_*(H)| **(a) **for the ^1^H^N ^shifts and of the uncorrected pressure coefficient |B_1_(N)| **(b) **for the ^15^N^H ^chemical shifts are plotted as function of the sequence position. The mean values of |B_1_*(H)| and |B_1_(N)| for the structured C-terminal and the unstructured N-terminal part of the protein are shown as solid lines, the mean values plus one standard deviation as dashed lines. The secondary structure is symbolized by arrows (β-strands) and lanyards (α-helices). P marks prolines, X other residues that are not visible or not assigned in the TROSY spectra.

**Figure 2:**
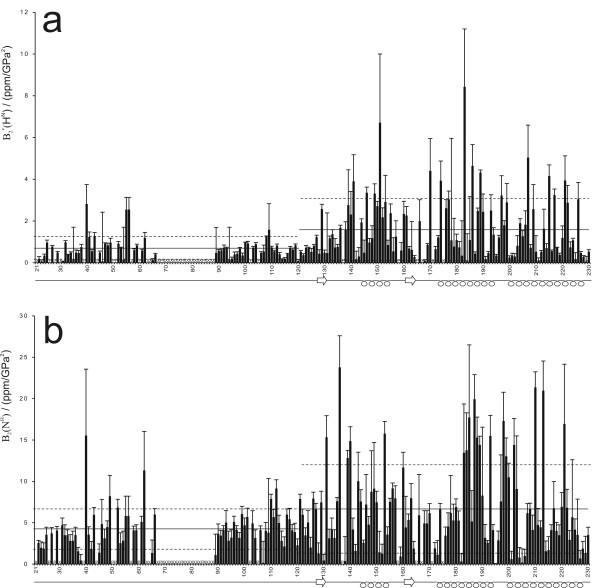
Second order pressure coefficients of the ^1^H^N ^and of the ^15^N^H ^chemical shifts taken from the TROSY-HSQC spectra of *hu*PrP(23–230). Spectra were measured at 293 K at 600 MHz proton frequency. The absolute values of the conformation dependent pressure coefficients |B_2_*(H)| **(a) **for the ^1^H^N ^shifts and of the uncorrected pressure coefficient |B_2_(N)| **(b) **for the ^15^N^H ^chemical shifts are plotted as function of the sequence position. The mean values of |B_2_*(H)| and |B_2_(N)| for the structured C-terminal and the unstructured N-terminal part of the protein are shown as solid lines, the mean values plus one standard deviation as dashed lines. The secondary structure is symbolized by arrows (β-strands) and lanyards (α-helices). P marks prolines, X other residues that are not visible or not assigned in the TROSY spectra.

The first order conformation dependent pressure |*B*_1_*(H)| for the ^1^H^N ^shifts and of the uncorrected pressure coefficient |*B*_1_*(N)*| of the ^15^N^H ^chemical shift as a function of the primary and the secondary structure are displayed in Fig. 1 for the full length protein *hu*PrP(23–230). Analogously, the second order coefficients |*B*_2_*(H)| and |*B*_2_*(N)*| are plotted as a function of the sequence position in Fig. 2. The mean first and second order pressure coefficients |*B*_1_*(H)|, |*B*_1_*(N)*|, |*B*_2_*(H)|, and |*B*_2_*(N)*| are 0.38 ppm/GPa, 3.35 ppm/GPa, 1.23 ppm/GPa^2^, and 5.70 ppm/GPa^2^.

A similar shift pattern is obtained for the truncated prion protein (data not shown) in the regions which are common to both proteins. However, the mean first and second order pressure coefficients |*B*_1_*(H)|, |*B*_1_*(N)*|, |*B*_2_*(H)|, and |*B*_2_*(N)*| are with 0.57 ppm/GPa, 3.35 ppm/GPa, 1.69 ppm/GPa^2^, and 7.29 ppm/GPa^2 ^significantly larger than in the full length protein. This is different when corresponding regions are compared since the pressure effects are much smaller in unfolded regions of the protein which contribute much more to the mean values in the full length protein. For the folded part of the full length protein one obtains mean values for <|*B*_1_*(H)|>, <|*B*_1_*(N)*|>, <|*B*_2_*(H)|>, and <|*B*_2_*(N)*|> 0.57 ppm/GPa, 3.33 ppm/GPa, 1.60 ppm/GPa^2^, and 6.62 ppm/GPa^2^, respectively.

If one takes the region common in the two proteins then the averages of the pressure coefficients are almost identical, that is the N-terminus does not significantly influence the average pressure response of the folded part. The correlation coefficient for the first order proton and nitrogen coefficients *B*_1_*(H) and *B*_1_*(N) *of the two proteins is 0.71 and 0.73, respectively, indicating also a quantitative agreement of the parameters in the two proteins. For the correlation coefficients of, *B*_2_*(H), and *B*_2_*(N) *one obtains with 0.34 and 0.63 smaller correlations in the two proteins. This is mainly due to the relatively large errors involved in the calculation of the Taylor pressure coefficients. In contrast, the direct correlation of the experimental chemical shift changes *Δδ(p) *(see Methods) is much higher. The mean value of these correlation coefficients for each residue of *hu*PrP(23–230) to its corresponding residue in *hu*PrP(121–230) is 0.86 for the amide protons and 0.93 for the amide nitrogen atoms.

**Figure 3:**
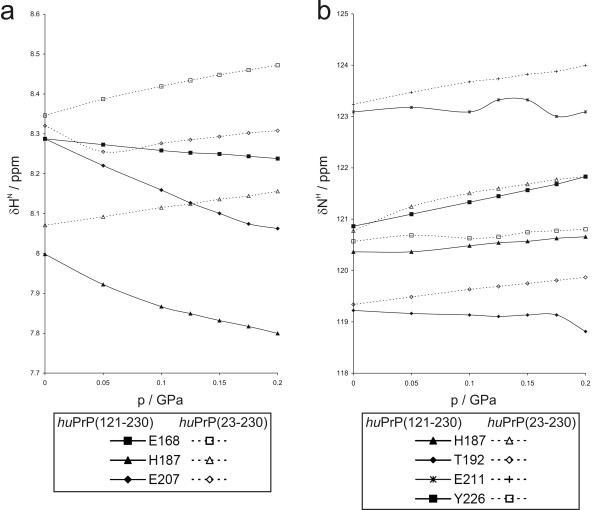
Differences in the pressure response in truncated and full length *hu*PrP. The pressure dependences of the ^1^H **(a) **and ^15^N chemical shifts δ **(b) **of selected amide groups are plotted for the truncated (solid lines) and the full-length PrP (dashed lines). Spectra were measured at 293 K at 600 MHz proton frequency.

Although the general pressure response of the folded core is very similar in the full length and the truncated *hu*PrP there are some residues where clearly different responses can be observed (Fig. 3). The chemical shift differences already existing for Glu168, His187, Thr192, Glu207, Glu211 and Tyr226 at ambient pressure become larger with increasing pressure.

In the structured core (amino acid 121–230) of full length and truncated *hu*PrP most residues shift with increasing pressure (Figs. 1 and 2). For identifying residues that behave differently to the majority of the peaks one can select those which display first or second order pressure coefficients which are larger or smaller than the average by a standard deviation (SD). Since large chemical shift changes are strongly correlated with large conformational changes, residues with pressure coefficients larger than average plus one SD are most probably involved in strong conformational changes. Accordingly, residues with pressure coefficients smaller than average minus one SD represent regions of small inferred conformational changes. The residues M129, F141, Q186, H187, T188, V189, K194, E196, E207, V210, E211, E219 and R220 display first order pressure coefficients |*B*_1_*(H)| and G131, I139, H140, C179, T183, T188, T191, K194, G195, N197, F198, T199, D202 and M205 |*B*_1_*(N)*| which are significantly larger than the average in this region (by one standard deviation) for *hu*PrP(121–230). The largest pressure induced chemical shift changes of amide proton resonances are observed in the loop between strand β1 (Y128 to G131) and helix α1 (D144 to M154), in the loop connecting helix α2 (N173 to K194) and α3 (E200 to R228), as well as in regions of helix α2 and α3. Generally, the differences of the pressure dependence of the chemical shifts along the protein sequence are much less pronounced for the amide nitrogen atoms than for the amide protons. The pressure-sensitive regions for both, ^1^H^N ^and ^15^N^H^, overlap quite well but not completely. Almost the same residues are also identified by the second order coefficients. The same is true for the residues with a very small pressure response, namely 129, 130, 148, 156, 174, 178, 182, 186, 187, 190, 192, 212, 219 and 221 (|*B*_1_*(N)*| smaller than the mean value minus one sigma) and 121, 122, 123, 126, 127, 128, 130, 133, 135, 138, 140, 142, 160, 164, 172, 177, 180, 184, 193, 198, 213, 222, 225, 226, 227, 228 (|*B*_1_*(H)| smaller than the mean value minus 0.75 sigma) in case of *hu*PrP(121–230). As has been stated above and has been quantified by the correlation analysis the pressure response of the amide nitrogen atoms of residues 121–230 of *hu*PrP(23–230) is very similar to that of *hu*PrP(121–230). In conclusion, *hu*PrP(121–230) is a good model for the study of pressure effects on the structured core of the whole protein.

The N-terminal part of the protein (amino acids 23 to 120) is assumed to be unstructured and thus should show only small pressure effects (Figs. 1 and 2). This is in general true, the mean first and second order pressure coefficients |*B*_1_*(H)|, |*B*_1_*(N)*|, |*B*_2_*(H)|, and |*B*_2_*(N)*| are with 0.18 ppm/GPa, 3.37 ppm/GPa, 0.68 ppm/GPa^2^, and 4.20 ppm/GPa^2 ^significantly smaller than in the folded part of the protein. However, a number of residues show pressure coefficients that are above the average. Surprisingly, this is also true for the amide proton shifts, where the pressure coefficients of random coil peptides [36] were subtracted. Notably, K27, S43, G46, G55, G56, K101, S103, K104 and M109 show linear pressure coefficients that deviate significantly from the expectation. For the quadratic coefficients this applies to G40, S43, G55, G56, N108 and M109. If the N-terminus would be completely unstructured, only pressure coefficients in the magnitude of the random coil peptide values would be expected.

### Pressure response of chemical shifts and related Gibbs free energies

**Figure 4:**
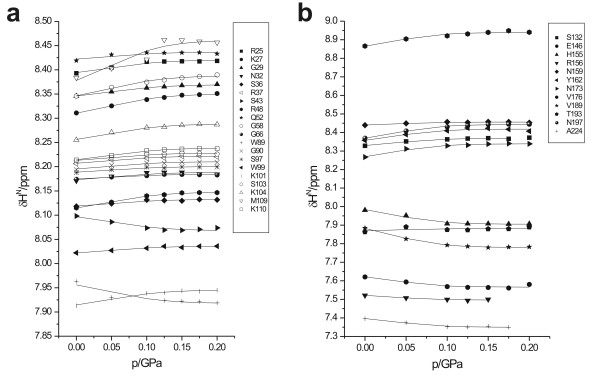
Pressure dependent population changes in fast exchange. The chemical shifts δ*H^N ^of the amide protons taken from a set of TROSY-HSQC spectra measured at 293 K are plotted as function of pressure p. The chemical shifts δ*H^N ^were corrected by the known pressure response of random-coil peptides (see Methods). All residues with a clearly asymptotic pressure dependence of the chemical shifts are shown. The lines represent the fit of the data to equation (2).**(a) **Data from N-terminal part of *hu*PrP(23–230) and **(b) **from the C-terminal structured part of *hu*PrP(121–230).

Some of the residues show strong non-linear pressure dependences of the chemical shifts together with an asymptotic behavior within the pressure range studied (Fig. 4) which would be typical for a two-state transition in fast exchange on the NMR-time scale. In this model, at ambient pressure two local conformational states N_1 _and N_2 _coexist that are sensed by the corresponding residues. At high pressure the local conformational equilibrium of the protein is completely shifted from the ground state to the low-lying excited state N_2_. The molar free energy (*ΔG*_0_) for this local transition can be derived from the chemical shift change by equation (2) (see Methods). As already discussed above for removing unspecific pressure effects from specific conformation dependent effects, prior to the calculation of the Gibbs free energies *ΔG*_0 _the pressure-induced chemical shift change as determined for the random coil standard peptides [36] was subtracted from the measured chemical shift values. Since that was only possible for the proton shifts only these were evaluated quantitatively. However, qualitatively the pressure dependence of the nitrogen chemical shifts followed the same rules.

For the residues of the structured core (*hu*PrP(121–230)) one obtains 3.0 ± 1.4 kJ/mol. The *ΔG*_0 _value for the pressure-induced transition of the N-terminal residues of *hu*PrP(23–230) is 3.0 ± 1.1 kJ/mol (Table 1). At ambient pressure about 71% of the protein are in the highest populated native "ground state" conformation, whereas 29% are in the second "excited" native conformation which is favored by applying pressure. From the fast exchange condition, a lower limit for the exchange correlation rate 1/τ_e _of 22 s^-1 ^can be estimated for the N_1_-N_2 _transition.

### Pressure response of cross peak volumes and the related Gibbs free energies

**Figure 5:**
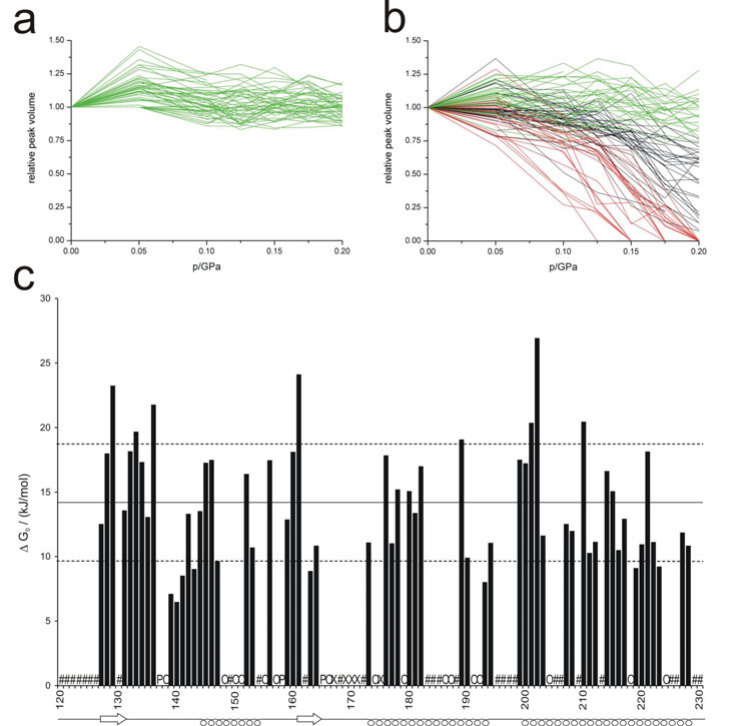
Pressure dependence of signal volumes in TROSY-HSQC spectra. The signal volumes are normalized to their volumes at ambient pressure and plotted as function of pressure. **(a) **Signal volumes from the N-terminus (residues 23–120) and **(b) **from the structured core (residues 121–230) are shown. Residues with no significant signal loss are highlighted green, disappearing signals are marked red. **(c) **Molar free energies at ambient pressure (Δ*G*_0_) for the N to I transition of *hu*PrP(121–230) calculated for individual residues assuming a slow exchange model. The mean value of Δ*G*_0 _(14.2 kJ/mol) is shown as solid line, the mean value plus/minus one standard deviation as dashed lines. Residues without a significant change in the signal volume are marked with #. O means, that the signal volume could not be determined due to heavy overlap of the peaks. P marks prolines, X other residues that are not visible or not assigned in the TROSY spectra.

At 200 MPa the amide proton resonances of residues Y128, G131, M134, R136, I139, F141, G142, S143, D144, Y150, R156, Q160, V161, Y163, N174, D178, I182, T199, E200, D202, V210, C214, I215, Q217 and E221 are not observable in case of *hu*PrP(121–230). Especially, the cross peak of Gly131 disappears already at 125 MPa, while residues Ile139, Phe141, Gln160, Val161, Tyr163 and Asp178 become undetectable at 150 MPa. These residues mainly cluster to the loop between the strand β1 and helix α1, near helix α3 and close to the β-sheet. In case of *hu*PrP(23–230) qualitatively the same process can be observed for the core region, however, since in the longer construct the resolution is reduced and becomes worse with increasing pressure only the disappearance of residues Gly131, Ile139, Phe141, Arg156 and Asp178 can be reliably confirmed. The N-terminal unstructured region displays a less pronounced chemical shift dependence and no significant volume change upon pressure (Fig. 5a). By releasing the pressure we observe the original spectra at ambient pressure again, thus the pressure-induced changes are completely reversible.

**Figure 6:**
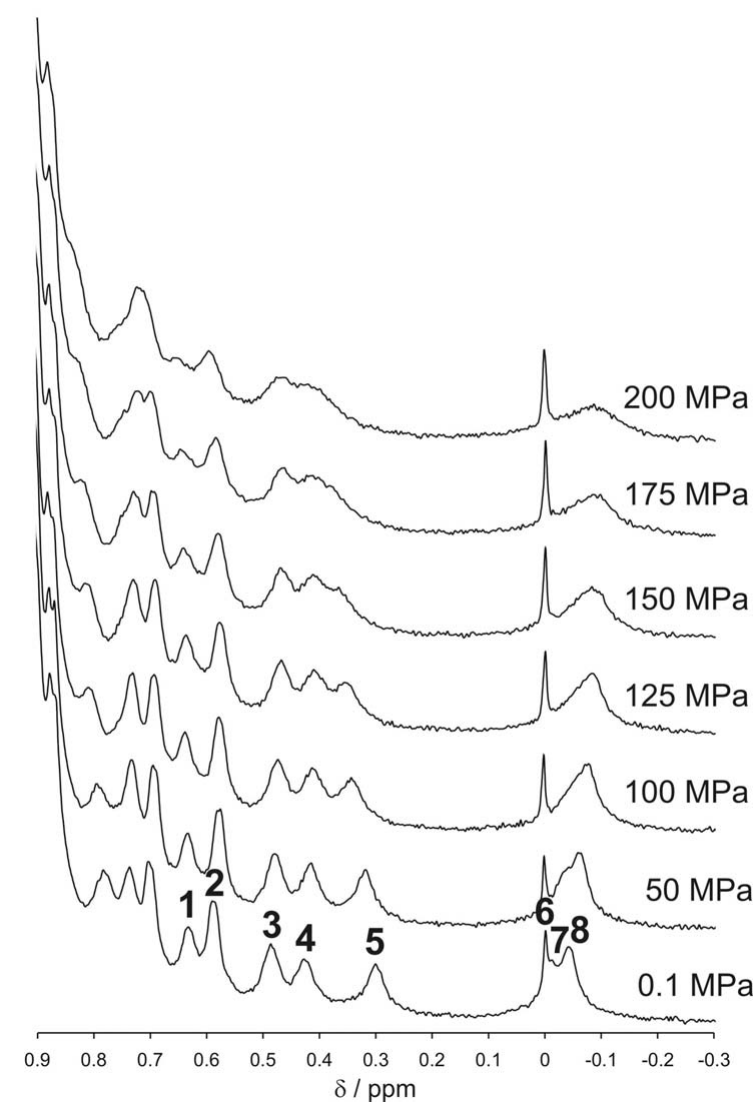
^1^H NMR spectra of *hu*PrP(23–230) recorded at 293 K, pH 4.8 and at different pressures. Resonances of selected protons are labeled: (**1**) Leu130 H^δ2 ^(**2**) Leu125 H^δ2 ^(**3**) Ile139 H^δ1 ^(**4**) Ile182 H^δ1 ^(**5**) Ile182 H^γ2 ^(**6**) DSS (internal reference) (**7**) Leu130 H^δ1 ^(**8**) Ile139 H^γ2^.

There are two possible explanations for this loss in intensity, increased loss of intensity during the INEPT-periods by increased average *T*_2_-relaxation and/or a two site exchange which is slow on the NMR-time scale and where the second signal would be too weak to be observable [37]. The increased transverse relaxation rate could be due to an exchange broadening or an increased effective molecular size by protein aggregation. The last mechanism could also lead to a disappearance of the second signal in the slow exchange regime. Under fast exchange conditions the increased transverse relaxation time can also be estimated from the broadening of the signals by pressure. The observed increase in line width by a factor 1.2 to 2 is too small to account for the observed loss in signal intensities if fast exchange conditions would prevail. Using the approximations derived by Maurer *et al*. [37] a maximum intensity decrease by 20% could follow from that additional, pressure dependent line broadening. For most of the residues under consideration the intensity loss during the polarization transfer process is considerably smaller, thus pointing towards a slow exchange process with an intermediate conformation. A line broadening of the same magnitude as observed for the amide groups is also observed in 1D-spectra for resonances of non-exchangeable protons. As an example the high-field shifted methyl resonances of Leu125, Leu130, Ile139 and Ile182 are shown in Fig. 6. As seen in the TROSY spectra, in the 1D spectra a certain number of peaks shows no or only weak line broadening, too. The line broadening is not caused by a change of the viscosity of water with pressure, since at 293 K the water viscosity shows only weak pressure dependence up to pressures above 200 MPa. Actually the viscosity even decreases till 80 MPa to about 98% and displays a moderate increase with increasing pressure above that. At about 140 MPa it reaches its original value again [38]. Thus, the origin of the line broadening might be due to conformational heterogeneity and/or exchange.

**Figure 7:**
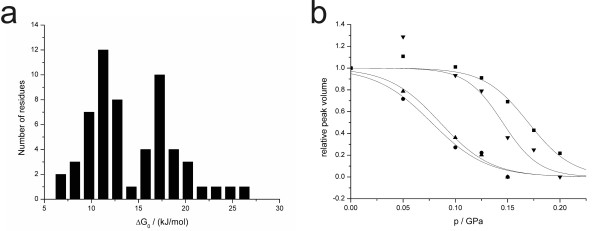
**(a) **Distribution of residue specific *ΔG*_0 _at 293 K for the transition from states N_1 _and N_2 _or N_2_only to state I_1 _and I_2_obtained from the pressure induced volume changes. **(b) **Pressure dependence of signal volumes in TROSY-HSQC spectra at 293 K with fit of the data by eq. 5. Typical residues of I_1 _(I139 ●, F141 ▲) and I_2 _(A133 ■, V210 ▼) are shown.

**Figure 8:**
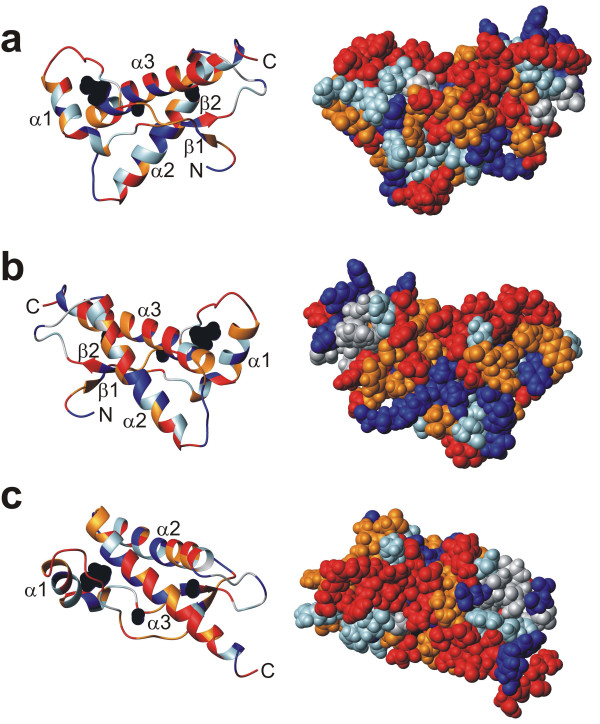
Pressure effects on *hu*PrP(121–230). **(a) **Ribbon and CPK representations of the NMR structure of *hu*PrP(121–230) (taken from PDB 1QM2) with molecular cavities added (dark spheres) defined by a probe radius of 0.12 nm. Residues showing no significant changes of the cross peak volumes are colored dark blue. Residues associated with *ΔG*_0_values characteristic for the N → I transition from group one (lower *ΔG*_0_) are depicted in red, from group two (higher *ΔG*_0_) orange. Regions where the signal volume could not be determined are colored light blue, residues not visible or assigned grey. **(b) **Same as (a) rotated by 180° around the y-axis. **(c) **Same as (a) rotated by 90° around the x-axis. Figure was prepared using *MOLMOL *[60].

Assuming two site model under slow exchange conditions the pressure induced changes of volumes and thus of the corresponding populations can be used to calculate a molar free energy (*ΔG*_0_) for every single amino acid residue in the polypeptide chain. The equilibrium constant *K *between the native state and the pressure populated intermediate can then be derived from the ratio of the peak volumes (see Methods). Fig. 5c shows the molar free energies at ambient pressure obtained from a fit of the data as a function of the primary structure of *hu*PrP(121–230). The secondary structure elements are indicated at the bottom. Due to signal overlap it was not possible to determine exact values for the cross peak intensities of every residue which leads to gaps in the residue specific determination of the pressure stability (marked with "O"). Residues which show no significant change of the signal intensity are marked with "*#*". The average for the molar free energies *ΔG*_0 _and their standard deviations are indicated in Fig. 5c by a solid and two broken lines. The mean of *ΔG*_0 _is 14.2 kJ/mol with a standard deviation of 4.5 kJ/mol. The distribution of free energies is depicted in Fig. 7a showing that the obtained energy distribution is clearly divided into two groups. Fig. 7b shows peak volumes and the *ΔG *fit of typical residues of both groups. In Fig. 8 the two groups are mapped on the structure of *hu*PrP(121–230). Group one encompasses 33 residues and has a *ΔG*_0 _of 10.8 ± 1.9 kJ/mol, the mean *ΔG*_0_of the 25 residues of group two is 18.6 ± 2.9 kJ/mol.

### Characterization of the folding intermediates

The pressure response of *hu*PrP consists of three different effects: [1] Small linear chemical shift changes corresponding to small first-order pressure coefficients, [2] Non-linear chemical shift changes which show for some residues a typical asymptotic behavior as it would be expected for a fast exchange between slightly differing conformations, and [3] a loss of signal volumes which occurs for the majority of the amino acid residues. A thorough analysis shows the appearance of at least two different conformational transitions between the ground state N_1_, the low-lying excited state N_2 _and the intermediate states [25,28,39]. At ambient pressure the chemical shift data allow us to postulate the states N_1 _and N_2_, and the analysis of the signal volume loss induced by pressure indicates additional intermediate states I_1 _and I_2_. Strong pressure responses are often associated with the existence of packing defects, or cavities in the direct neighborhood. The cavities calculated from the PDB deposited structure (code 1QM2) are also shown in Fig. 8. The found cavity at this position is surrounded by acidic side chains thus leading to a hydrophilic pore. Close to helices α2 and α3 similar packing defects are also seen.

**Figure 9:**
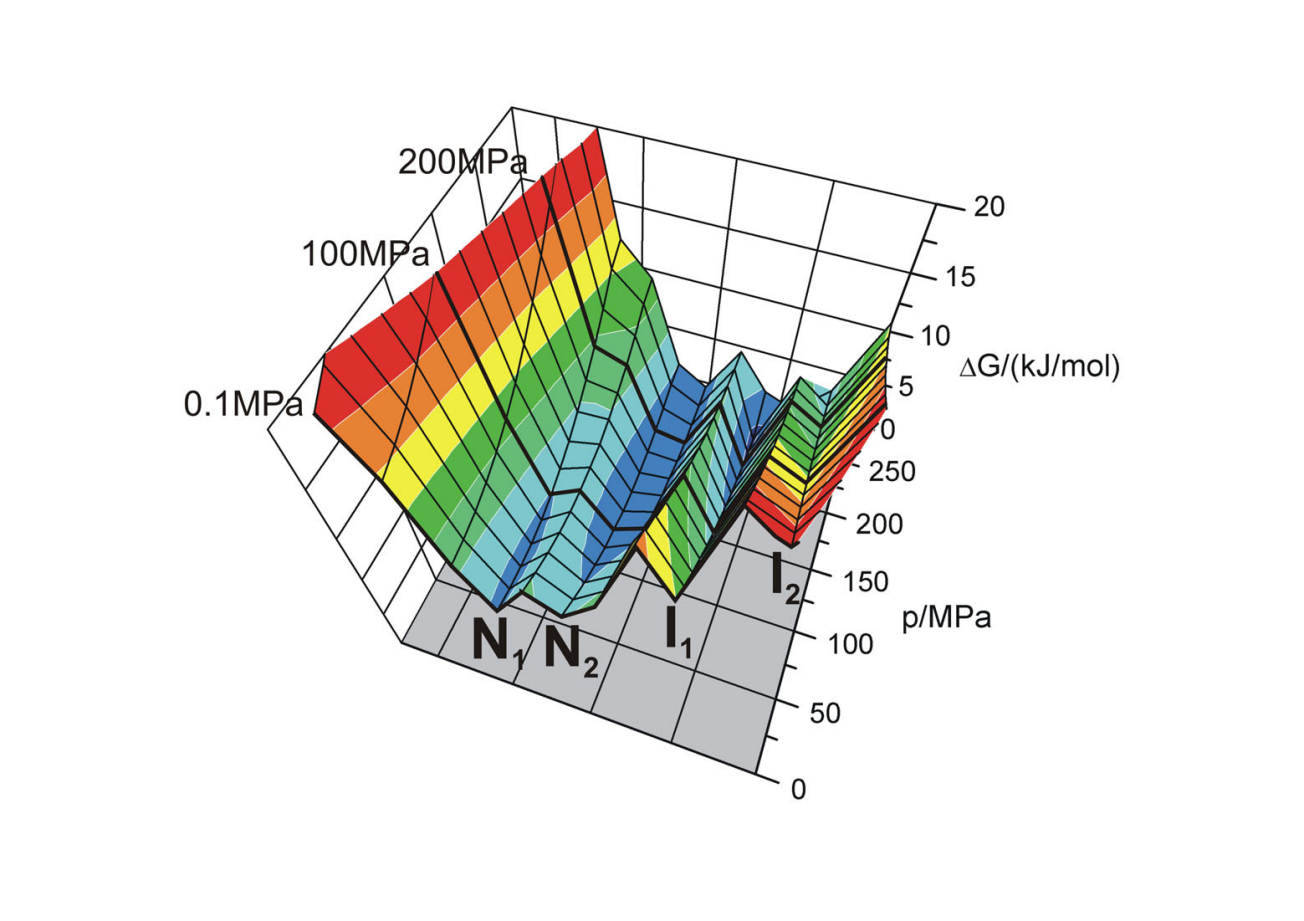
Schematic view of the free energy landscape of the human prion protein as a function of pressure. The molar free energy differences ΔG_i _= G_i_(p)-G_N_(p) of the four main conformations N_1_, N_2 _and I_1 _and I_2 _are depicted as function of the pressure p at constant temperature T of 293 K. In this representation as ground state N the state N_1 _was assumed (but see discussion). Note that the abscissa represents a generalized coordinate in the conformational space that only schematically depicts the conformational states.

The pressure dependent energy landscape of the human prion protein is shown in Fig. 9 schematically. At ambient pressure two native states N_1 _and N_2 _as well as the energetic unfavorable excited intermediate states I_1 _and I_2 _are visible. With increasing pressure the energy profile changes. At the onset of pressure N_2 _becomes the energetically lowest and therefore highest populated conformation. At very high pressures the intermediate conformations I_1 _and I_2 _are strongly favored.

## Discussion

A key point of the prion only hypothesis has been finally proven by different groups [8,14,40] showing that it is possible to produce *in vitro *conformations of the prion protein which can confer infectivity. This conformation is generated by a seeding process which is so potent, that even sequence-based barriers that prevent cross-seeding between prion proteins from different species can be bypassed *in vitro*. Such a template-induced adaptation process is leading then to the emergence of new strains of prion fibrils [12,41,42]. It is obvious, that the pathway of prion protein assembly into amyloid and possibly into infectious protein implies a whole variety of subconformations and is extremely complex [43].

The investigation of multiple misfolding pathways of the prion protein is indispensable to pinpoint down the regions involved in the protein conversion from PrP^C ^to PrP^Sc^. Folding or unfolding intermediates are usually created by use of denaturing agents. An elegant alternative to this is the application of hydrostatic pressure which allows to stabilize and study intermediate forms in the folding pathway of PrP [33]. Pressure can also induce scrapie-like prion protein misfolding and amyloid fibril formation as has been shown by Lange and co-workers [30]. Torrent *et al*. [30] showed an irreversible aggregation of *Syrian hamster *prion protein *Sha*PrP(90–231) above 450 MPa, and incubation of *Sha*PrP(90–231) at 600 MPa overnight led to the formation of amyloid fibrils, whereas pressures up to 200 MPa led to reversible effects and recovery of the original structure after pressure release [29]. NMR spectroscopy is the only generally applicable method to monitor pressure-induced structural changes at the atomic level in solution. Our current setup allows us to monitor such changes between 0.1 and 200 MPa. Here all pressure-induced structural changes were reversible as also be observed by NMR in the hamster prion protein [34]. However, a detailed comparison at an atom to atom level with our data is not possible since Kuwata et al. used a less sophisticated evaluation method, especially in that study it was not investigated if the native and intermediate state can be differentiated in substates with different free energies.

### Conformational states in proteins detected by variation of pressure

Application of equations (2) to (5) allows the calculation of individual Δ*G*_0 _and Δ*V *values for all atoms in the molecule. When more than one parameter can be used as in our case the chemical shift change and the peak volume change more than one Δ*G*_0 _and Δ*V *value can be calculated for the structural transition observed by a single amide group in the protein. The question arises what the physical meaning of these values may be. In principle, Δ*G*_0 _and Δ*V *are global quantities that describe the difference of the Gibbs free energies and partial volumes of a state X_i _to an arbitrary state X_1 _of the system. Since the number of possible states of the system (that is the number of conformational states of the protein and the solvent molecules) is infinite one has to restrict to a smaller number of global states for obtaining meaningful results. In general one can separate the states of the bulk water from the rest of the system. Since pressure influences strongly the water shell close to the protein the effects on the shell and the protein conformation itself are difficult to separate.

Our analysis of N amide cross peak volumes and 2N pressure induced ^1^H and ^15^N chemical shift changes gives us information about 6N different conformational states when the individual parameters follow a two-site exchange model. In case of multiple site exchange even more (sub)states would be expected. The primary evaluation of our shift and volume changes is done in this way. The same has been done by Kuwata *et al*. for the hamster prion protein but only volume changes were taken into account [34].

However, the structural substates in a protein-water system are usually coupled; the obtained atom specific Δ*G*_0 _and Δ*V *values are in general not to be interpreted locally since large contributions are due to structural differences of other parts of the molecule compared to the ground state (or the set of closely spaced low-energy states). As a further complication the calculation of the differences as done here must not necessarily refer to the same ground state.

After correction for unspecific "random coil" pressure induced shifts, we can group the Δ*G*_0 _and Δ*V *values in four main classes. This means in a minimal description of the system the substates X_i _are reduced to the combination of two "native" states N_1 _and N_2 _and two "intermediate" states I_1 _and I_2_. These states are primarily defined in the data evaluation as localized structural states and no information is available about the structural states of not directly considered other parts of the protein. Especially, in the data evaluation of the signal volume changes defining the intermediate states a localized native structural state has to be assumed. Since for some of the residues undergoing volume changes also shift changes corresponding to the N_1 _to N_2 _transition are observed, it seems plausible to assume that this transition is independent of the intermediate states and occurs in the whole protein. The calculated Δ*G*_0 _values then would refer to a state N where the other parts of the molecules are in the structural state N_1 _or N_2_. The simplest picture would assume an ordered reaction scheme of conformational states S.

N_1 _⇆ N_2 _⇆ I_1 _⇆ I_2_

The quality of the data does not allow distinguishing between this model and more complicated possible models. The corresponding energy surface has been depicted schematically in Fig. 9. The abscissa here is a generalized arbitrary coordinate which represents the structural states of the proteins only schematically.

Since the data can be explained sufficiently well with four states only, we restrict in the following discussion on the existence of these four states. However, we cannot exclude the existence of additional structural states with indistinguishable free energies. The pressure dependences of the chemical shifts indicate the existence of a second native conformation of *hu*PrP, which is favored under high pressure and is in fast conformational exchange with the "ground state". At ambient pressure 71% of the protein is in the main native state N_1 _and 29% are in the "excited" native conformation N_2_. The low energy difference (*ΔG*_0 _= 3.0 kJ/mol) and the relatively small chemical shift differences between N_1 _and N_2 _implies, that only slight structural changes occur in the N_1 _↔ N_2 _transition. The state I_1 _and I_2 _are derived from the analysis of the volume changes of those resonances that are too broad to be observable in one of the states I. At ambient pressures only less than 1% exists in I_1 _or I_2_. At 200 MPa most of the protein is in state I_1 _or I_2_.

### Structural changes under high pressure

The N-terminal region of *hu*PrP(23–230) is known to show no well-defined three-dimensional structure at the used buffer conditions. An analysis of the chemical shifts corrected for neighbor effects [44] indicates no well-defined regions where secondary structure is induced by pressure. Nevertheless, the complete N-terminus has a tendency towards positive chemical shift values.

After subtracting the random-coil pressure coefficients within the limits of error vanishing first and second order pressure coefficients are expected for those parts of the structure that are in a random-coil conformation. Unfortunately, random-coil values do only exist for protons [36] but not for ^15^N. Therefore, only the proton shifts can be interpreted in detail. In the presumably unstructured part (amino acids 23 to 120) the expectation values of *B*_1_* and *B*_2_* are with 0.11 ppm/GPa and 0.68 ppm/GPa^2 ^close to zero (see Results). Most interestingly, the range of amino acids 90 to 170 where two-dimensional electron crystallography and molecular dynamics simulations [45,46] predict β-sheet formation after polymerization displays a high variety of pressure sensitivity. The strands β1 and β2 seem to be very insensitive to the application of high pressure while the loop and the helix α1 in between these two strands exhibit some of the most pronounced changes in structure we observed by applying high pressure.

As indicated by the pressure induced chemical shift changes there are two native conformations N_1 _and N_2 _in fast conformational exchange, which coexist even at ambient pressure. The relatively small difference of the molar free enthalpies of both states as well as the rather small chemical shift changes indicate, that the N_1 _↔ N_2 _transition is only associated with a small structural rearrangement. Especially the very small chemical shift differences show that N_1 _and N_2 _adopt very similar structures. Unfortunately, a complete structural characterization of N_2 _cannot be performed by the available data.

The pressure dependent reduction of cross peak volumes in the TROSY-HSQC-spectra was associated with the N ↔ I transition. They can be due to increased T_2_-losses during the INEPT polarization transfer periods and/or a slow exchange process where the resonances of state I are broadened beyond detection [37]. In principle mainly two mechanisms can be responsible for an increased T_2_, an increase of the rotational correlation time by protein-protein interaction and exchange broadening. In one-dimensional proton NMR spectra we observe a nearly general line broadening of approximately a factor two which could indicate a formation of dimers at higher pressure. In the TROSY-HSQC spectra also an additional line broadening with pressure is observed which varies locally but is clearly smaller than the factor of two. Additional line broadenings in the TROSY-spectra are mainly associated with chemical exchange processes. The observed increase of the transverse relaxation rates cannot explain the observed reduction of cross peak volumes as the only factor (see Results). Therefore, as additional factor a reduction of the peak volume by a slow exchange process is required as it has been done also for the hamster prion protein by Kuwata *et al*. [34]. In the absence of exchange broadening the cross peak volume change with pressure can be described by equations (4) and (5). In the presence of a moderate additional exchange these equations still provide a good approximation but the presence of exchange broadening would lead to too large estimates of the apparent *ΔG*_0 _values.

Within the limits of error the cross peak volume changes can be associated with two folding intermediates. In the folded core of the prion protein the region around positions Ile139, His140, and Phe141 shows rather low *ΔG*_0 _values that may indicate a larger exchange contribution. This may be interpreted as a premature local melting which correlates with a packing defect in this region (Fig. 8). A non-perfect stacking of side-chain and main-chain atoms leads to a less restricted peptide main chain in this area allowing a high degree of conformational freedom and thus conformational variability/heterogeneity. As pointed out by Pratt and co-workers [47] and has been observed experimentally by high-pressure NMR spectroscopy on RalGDS by us [25], pressure-induced unfolding is caused by the penetration of water into the hydrophobic core due to the dissociation of electrostatic bonds and solvation of hydrophobic residues. The sidechain resonance of the Ile139 methyl proton exhibits the most pronounced up field shift observed in the human prion protein indicating a strong interaction with the hydrophobic core of this protein.

Single low *ΔG*_0 _values are also found in the helical region encompassing α2 and α3 which is in agreement with the finding that helices are more prone to pressure than extended structures like β-strands. In a similar study Kuwata *et al*. [34] investigated in a high-pressure NMR study the *Syrian hamster Sha*PrP(90–231). They found the most pronounced effects in the helical region encompassing the helices α2 and α3 implying a complete unfolding of these two helices in a slow exchange process. In addition, our data show the occurrence of a pressure-stabilized "excited" state N_2_. The existence of that state was not taken into account in their data analysis. Subsequently we find a slow exchange process to two intermediate conformations, which are characterized by a melting of the region around Ile139-Phe141 and a partial melting of the helical region encompassing α2 and α3. Our data suggest a dual mechanism for the structural changes under pressure at least in the human prion protein. Especially the observation in intermediate I_2 _that the most stable region of the human prion protein is localized in the region of the two helices where the disulfide bridge is located corresponds very well with the hydrogen protection measurements by other groups [48,49].

Vanik *et al*. [16] adopted the Y145 Stop variant for *in vitro *specific seeding of amyloid fibrils. In particular, they found that mouse or hamster PrP-specific amino acid substitutions at position 138 and 139 are sufficient to change essential amyloidogenic properties of *hu*PrP(23–144), with the mutant proteins adopting seeding specificities of PrP corresponding to different species. Vice versa, M138I/M139I *Sha*PrP(23–144) adopted the properties of human PrP(23–144) [16]. Our results show that the importance of individual amino acids in PrP as determinants of the species barriers are due to their local conformational variability which can be highlighted by applying high pressure and monitored on an atomic scale through NMR spectroscopy. Especially, local clusters of very low stability found in our experiments are prone to change their conformation upon interaction with the infectious conformer PrP^Sc^. Indeed, permissible first entry-points of the scrapie isomer need a certain degree of local conformational freedom to initiate the refolding of the cellular isomer to an infectious one. Thus, the species and transmission barrier is conserved through the differences in the conformational freedom of local areas in the protein structure which can vary substantially with differing solution conditions necessary for the proteolytic degradation of proteins in the cell.

### Structural states of N-terminal parts of the *hu*PrP

The N-terminal part of *hu*PrP(23–230) exhibits regions with chemical shift responses that are not typical for random-coil peptides. Their first and/or second order pressure coefficients deviate significantly from the average (by more than a standard deviation). This is best visible for the proton shift coefficients since they could be corrected by subtracting the published random-coil values [36]. One can define four regions where at least one of the coefficients deviates significantly from the average, namely region A encompassing K27, region B encompassing G40, S43 and G46, region C encompassing G55 and G56 and region D with K101, S103, K104, N108 and M109. The data indicate that these regions have a non-random population of structural states that coexist in solution and show a characteristic change of populations with pressure. Region C is located within the three octapeptide repeats which are known to adopt a defined structure upon binding of copper [35]. Unfortunately, the amino acids of the three octapeptide repeats could not be resolved individually in the spectra. They seem to have a similar pressure response, too. Region D is in the region Kuwata *et al*. previously discussed the existence of transient helices [34]. This is in good agreement with our observations of pressure responses typical for structured proteins. Interestingly, there is an overlap between region B and C of the pressure-sensible residues and a possible pH-dependent aggregation site (residues 45–66) as described by Ralph Zahn [35]. The observed pressure-sensitivity emphasizes the importance of this region.

The probability that some residues of the unfolded part of PrP are in the time average in contact with the surface of the folded part is quite high and was discussed by Zahn *et al*. [19]. If this would be true it could also influence the pressure response of the folded core in full length PrP. Indeed, such a differential response is observed clearly for Glu168, His187, Thr192, Glu207, Glu211 and Tyr226. They show also different chemical shifts in truncated and full length PrP. From C^α ^chemical shift differences in the full length and the truncated PrP Zahn *et al*. [19] identified as possible interaction regions His187 to Thr193 and Glu219 to Tyr226. The differences in the pressure response observed may indicate a transient interaction between the region D that is closest in sequence to the core and the surface around amino acids mentioned above.

## Conclusion

High-pressure NMR spectroscopy indicates that the folded core of the human prion protein occurs in two structural states N_1 _and N_2 _in solution associated with rather small differences in free enthalpies (3.0 kJ/mol). At atmospheric pressure approximately 29% of the protein are already in the pressure favored conformation N_2_. There is a second process representing two possible folding intermediates I_1 _and I_2 _with average free enthalpy differences of 10.8 kJ/mol and 18.6 kJ/mol respectively, which could represent a preaggregation state of the protein. Freezing out of such subpopulations by fixing them, e.g. with antibodies added or co-expressed in transgenic animals [50,51], has led to animal strains resistant against the development of prionosis. In this sense such local areas of high conformational variability/heterogeneity highlighted through high pressure NMR spectroscopy are indeed a first drug target for preventing the conversion into toxic intermediates able to transform into amyloid fibrils.

## Methods

### Protein expression and sample preparation

Recombinant human prion protein *hu*PrP(23–230) (residues 23 to 230) and *hu*PrP(121–230) (residues 121 to 230) was prepared as described previously [19,52]. Note that due to cloning artifacts the used recombinant proteins have two additional residues at the N-terminus (G21 and S22 in case of *hu*PrP(23–230), G119 and S120 in case of *hu*PrP(121–230). For the NMR experiments a 1.1 mM solution of ^15^N-enriched *hu*PrP(23–230) and a 1.2 mM solution of ^15^N-enriched *hu*PrP(121–230) each in 10 mM sodium acetate buffer pH 4.8 was used. The measurements were performed in ^1^H_2_O with 8% ^2^H_2_O added. The samples contained 0.1 mM of 2,2-dimethyl-2-silapentane-5-sulfonate (DSS) as internal reference.

### High pressure NMR measurements

All NMR experiments were carried out on a Bruker DRX 600 spectrometer, operating at 600 MHz proton resonance frequency. To apply high pressure we used an on-line variable pressure cell system [53,54] with a sapphire capillary of 1.72 mm inner and 3.14 mm outer diameter [55,56]. Spectra were recorded at hydrostatic pressures of 0.1, 50, 100, 125, 150, 175 and 200 MPa. At a temperature of 293 K and different pressures one-dimensional ^1^H spectra and two-dimensional ^1^H-^15^N-TROSY spectra were acquired. The TROSY spectra [57] had a digital resolution of 2048 * 512 points and a frequency width of 7184 * 1825 Hz. All spectra were recorded and processed using the *XWINNMR *package from Bruker. *AUREMOL *[58,59] was used for peak picking, signal volume integration and analysis of the spectra.

### Analysis of the chemical shifts

The spectra were assigned on basis of the assignment at ambient pressure [19]. The chemical shift values of the amide protons and nitrogen atoms in the TROSY spectra under the influence of pressure were fitted to equation (1), where *δ*_0 _is the chemical shift at ambient pressure *p*_0_. The first order pressure coefficient *B*_1 _and the second order pressure coefficient *B*_2 _of each amide proton were corrected by subtracting the pressure coefficients of the standard peptide (Gly-Gly-X-Ala) of the corresponding amino acid as published by Arnold *et al*. [36]. For glutamate the coefficients of the C-terminal methylated peptide (Gly-Gly-Glu-Ala-methyl) were used (*B*_1 _0.53 ppm/GPa, *B*_2 _-0.98 ppm/GPa^2^) [56]. The corrected coefficients are referred as *B*_1_* and *B*_2_*.

*δ*(*p*,*T*_0_) = *δ*_0_(*p*_0_,*T*_0_) + *B*_1_(*p*_0_,*T*_0_)(*p *- *p*_0_) + *B*_2_(*p*_0_,*T*_0_)(*p *- *p*_0_)^2 ^    (1)

If there is an equilibrium between two conformations of the protein that is in fast exchange on the time scale of the NMR experiment, the observed chemical shifts are the weighted average of the chemical shifts *δ*_1 _and *δ*_2 _of the two states as described by equation (2), with an equilibrium constant for the conformational exchange K=N2N1
 MathType@MTEF@5@5@+=feaafiart1ev1aaatCvAUfKttLearuWrP9MDH5MBPbIqV92AaeXatLxBI9gBaebbnrfifHhDYfgasaacH8akY=wiFfYdH8Gipec8Eeeu0xXdbba9frFj0=OqFfea0dXdd9vqai=hGuQ8kuc9pgc9s8qqaq=dirpe0xb9q8qiLsFr0=vr0=vr0dc8meaabaqaciaacaGaaeqabaqabeGadaaakeaacqWGlbWscqGH9aqpdaWcaaqaaiabd6eaonaaBaaaleaacqaIYaGmaeqaaaGcbaGaemOta40aaSbaaSqaaiabigdaXaqabaaaaaaa@336F@.

δ=11+Kδ1K1+Kδ2=δ1+δ2exp⁡(−ΔGRT)1+exp⁡(−ΔGRT)=δ1+(δ2−δ1)11+exp⁡(ΔGRT)     (2)
 MathType@MTEF@5@5@+=feaafiart1ev1aaatCvAUfKttLearuWrP9MDH5MBPbIqV92AaeXatLxBI9gBaebbnrfifHhDYfgasaacH8akY=wiFfYdH8Gipec8Eeeu0xXdbba9frFj0=OqFfea0dXdd9vqai=hGuQ8kuc9pgc9s8qqaq=dirpe0xb9q8qiLsFr0=vr0=vr0dc8meaabaqaciaacaGaaeqabaqabeGadaaakeaaiiGacqWF0oazcqGH9aqpdaWcaaqaaiabigdaXaqaaiabigdaXiabgUcaRiabdUealbaacqWF0oazdaWgaaWcbaGaeGymaedabeaakmaalaaabaGaem4saSeabaGaeGymaeJaey4kaSIaem4saSeaaiab=r7aKnaaBaaaleaacqaIYaGmaeqaaOGaeyypa0ZaaSaaaeaacqWF0oazdaWgaaWcbaGaeGymaedabeaakiabgUcaRiab=r7aKnaaBaaaleaacqaIYaGmaeqaaOGagiyzauMaeiiEaGNaeiiCaaNaeiikaGIaeyOeI0YaaSaaaeaacqGHuoarcqWGhbWraeaacqWGsbGucqWGubavaaGaeiykaKcabaGaeGymaeJaey4kaSIagiyzauMaeiiEaGNaeiiCaaNaeiikaGIaeyOeI0YaaSaaaeaacqGHuoarcqWGhbWraeaacqWGsbGucqWGubavaaGaeiykaKcaaiabg2da9iab=r7aKnaaBaaaleaacqaIXaqmaeqaaOGaey4kaSYaaeWaceaacqWF0oazdaWgaaWcbaGaeGOmaidabeaakiabgkHiTiab=r7aKnaaBaaaleaacqaIXaqmaeqaaaGccaGLOaGaayzkaaWaaSaaaeaacqaIXaqmaeaacqaIXaqmcqGHRaWkcyGGLbqzcqGG4baEcqGGWbaCcqGGOaakdaWcaaqaaiabgs5aejabdEeahbqaaiabdkfasjabdsfaubaacqGGPaqkaaGaaCzcaiaaxMaadaqadiqaaiabikdaYaGaayjkaiaawMcaaaaa@7BEC@

The free energy at ambient pressure *ΔG*_0 _can be determined by fitting the data to equation (3), which represents the first terms of a Taylor expansion of Δ*G *as function of pressure *p *at constant temperature *T*. Here, *ΔV *is the partial molar volume and *Δβ *the compressibility. For our calculations of the *ΔG*_0 _values we neglected the quadratic term in equation (3).

ΔG=ΔG0+ΔV(p−p0)+12Δβ(p−p0)2     (3)
 MathType@MTEF@5@5@+=feaafiart1ev1aaatCvAUfKttLearuWrP9MDH5MBPbIqV92AaeXatLxBI9gBaebbnrfifHhDYfgasaacH8akY=wiFfYdH8Gipec8Eeeu0xXdbba9frFj0=OqFfea0dXdd9vqai=hGuQ8kuc9pgc9s8qqaq=dirpe0xb9q8qiLsFr0=vr0=vr0dc8meaabaqaciaacaGaaeqabaqabeGadaaakeaacqGHuoarcqWGhbWrcqGH9aqpcqGHuoarcqWGhbWrdaWgaaWcbaGaeGimaadabeaakiabgUcaRiabgs5aejabdAfawnaabmGabaGaemiCaaNaeyOeI0IaemiCaa3aaSbaaSqaaiabicdaWaqabaaakiaawIcacaGLPaaacqGHRaWkdaWcaaqaaiabigdaXaqaaiabikdaYaaacqGHuoariiGacqWFYoGydaqadiqaaiabdchaWjabgkHiTiabdchaWnaaBaaaleaacqaIWaamaeqaaaGccaGLOaGaayzkaaWaaWbaaSqabeaacqaIYaGmaaGccaWLjaGaaCzcamaabmGabaGaeG4mamdacaGLOaGaayzkaaaaaa@4EFB@

### Analysis of the signal volumes

Is there a conformational exchange which is slow relative to the time scale of the NMR experiment, the signal volume of one state is proportional to its population. Assuming a two-state transition for the change of the conformational equilibrium with increased pressure, the equilibrium constant *K *can be calculated from the relative cross-peak intensities of the TROSY spectra by equation (4) [34]. *I(p) *is the relative cross-peak intensity at pressure *p *and *I*_0 _is the one, when 100% of the protein are in the native state. With this one obtains the free energy *ΔG *associated with the pressure-induced conformational change by equation (5). The free energy at ambient pressure Δ*G*_0 _is defined by equation (3) and can be obtained from a three parameter fit with *I*_0_, Δ*V*_0 _and Δ*G*_0 _neglecting the second order term in p.

K=1−I(p)I0I(p)I0     (4)
 MathType@MTEF@5@5@+=feaafiart1ev1aaatCvAUfKttLearuWrP9MDH5MBPbIqV92AaeXatLxBI9gBaebbnrfifHhDYfgasaacH8akY=wiFfYdH8Gipec8Eeeu0xXdbba9frFj0=OqFfea0dXdd9vqai=hGuQ8kuc9pgc9s8qqaq=dirpe0xb9q8qiLsFr0=vr0=vr0dc8meaabaqaciaacaGaaeqabaqabeGadaaakeaacqWGlbWscqGH9aqpdaWcaaqaaiabigdaXiabgkHiTmaalaaabaGaemysaK0aaeWaceaacqWGWbaCaiaawIcacaGLPaaaaeaacqWGjbqsdaWgaaWcbaGaeGimaadabeaaaaaakeaadaWcaaqaaiabdMeajnaabmGabaGaemiCaahacaGLOaGaayzkaaaabaGaemysaK0aaSbaaSqaaiabicdaWaqabaaaaaaakiaaxMaacaWLjaWaaeWaceaacqaI0aanaiaawIcacaGLPaaaaaa@413F@

I(p)=I01+exp⁡(−ΔGRT)     (5)
 MathType@MTEF@5@5@+=feaafiart1ev1aaatCvAUfKttLearuWrP9MDH5MBPbIqV92AaeXatLxBI9gBaebbnrfifHhDYfgasaacH8akY=wiFfYdH8Gipec8Eeeu0xXdbba9frFj0=OqFfea0dXdd9vqai=hGuQ8kuc9pgc9s8qqaq=dirpe0xb9q8qiLsFr0=vr0=vr0dc8meaabaqaciaacaGaaeqabaqabeGadaaakeaacqWGjbqsdaqadiqaaiabdchaWbGaayjkaiaawMcaaiabg2da9maalaaabaGaemysaK0aaSbaaSqaaiabicdaWaqabaaakeaacqaIXaqmcqGHRaWkcyGGLbqzcqGG4baEcqGGWbaCcqGGOaakcqGHsisldaWcaaqaaiabgs5aejabdEeahbqaaiabdkfasjabdsfaubaacqGGPaqkaaGaaCzcaiaaxMaadaqadiqaaiabiwda1aGaayjkaiaawMcaaaaa@4568@

### Analysis of pressure dependent shift correlations

When the chemical shift changes in two samples A and B (e. g. the same spin in two constructs of a protein or the hydrogen and nitrogen atoms of an amide group) are governed by the same physical process that can be approximated by a two-site exchange then the chemical shift changes can be described by the same free energy *ΔG*. In this case the pressure dependent chemical shift changes *Δδ*^*A*^and *Δδ*^*B*^

Δ*δ*^*A *^= *δ*^*A*^(*p*) - *δ*^*A*^(*p*_0_) = (*δ*_2_^*A *^- *δ*_1_^*A*^)*f*(*p*)     (6a)

Δ*δ*^*B *^= *δ*^*B*^(*p*) - *δ*^*B*^(*p*_0_) = (*δ*_2_^*B *^- *δ*_1_^*B*^)*f*(*p*)     (6b)

follow the linear relation



## Authors' contributions

WK and NK contributed equally to the manuscript. WK carried out the high pressure NMR experiments and the first analysis of the data and wrote a first version of the manuscript. NK finalized the data analysis, further developed the manuscript and created the manuscript figures. RZ provided structural data on PrP at ambient pressure. HRK conceived the study, participated in its design and was strongly involved into drafting the manuscript. All authors read and approved the final manuscript.

## Figures and Tables

**Table 1:** Gibbs free energies and specific volumes^a^

**Domain**	**Transition**	***K***_0_	**Δ*G*_0 _[kJ mol^-1^]**	**Δ*V*_0 _[ml mol^-1^]**	**Δ*V*_0 _[nm^3^]**
*hu*PrP(23–230)					
amino acid 23–120	N_1 _▶ N_2_	0.292	3.0 ± 1.1	-63 ± 11	-0.10 ± 0.02
	N ▶ I	n. d.	n. d.	n. d.	n. d.
amino acid 121–230	N_1 _▶ N_2_	0.300	2.9 ± 1.3	-79 ± 15	-0.13 ± 0.02
	N ▶ I	n. d.	n. d.	n. d.	n. d.

*hu*PrP(121–230)					
amino acid 121–230	N_1 _▶ N_2_	0.292	3.0 ± 1.4	-86 ± 17	-0.14 ± 0.03
	N ▶ I_1_	0.012	10.8 ± 1.9	-66 ± 26	-0.11 ± 0.04
	N ▶ I_2_	0.0005	18.6 ± 2.9	-125 ± 32	-0.21 ± 0.05

^a ^Data were measured at 293 K and represent the average of the residues in the corresponding domains. *K*_0 _and Δ*G*_0 _are the values calculated for 293 K and 0.1 MPa.
